# Developmental mosaicism underlying *EGFR*-mutant lung cancer presenting with multiple primary tumors

**DOI:** 10.1038/s43018-024-00840-y

**Published:** 2024-10-15

**Authors:** Risa Burr, Ignaty Leshchiner, Christina L. Costantino, Martin Blohmer, Tilak Sundaresan, Justin Cha, Karsen Seeger, Sara Guay, Brian P. Danysh, Ira Gore, Raquel A. Jacobs, Kara Slowik, Filippo Utro, Kahn Rhrissorrakrai, Chaya Levovitz, Jaimie L. Barth, Taronish Dubash, Brian Chirn, Laxmi Parida, Lecia V. Sequist, Jochen K. Lennerz, Mari Mino-Kenudson, Shyamala Maheswaran, Kamila Naxerova, Gad Getz, Daniel A. Haber

**Affiliations:** 1 Krantz Family Center for Cancer Research, Massachusetts General Hospital Cancer Center and Harvard Medical School, Charlestown, MA USA; 2 Cancer Program, Broad Institute of MIT and Harvard, Cambridge, MA USA; 3 Department of Medicine, Boston University, Boston, MA USA; 4 Department of Surgery, Massachusetts General Hospital and Harvard Medical School, Boston, MA USA; 5 Department of Genetics, Harvard Medical School, Boston, MA USA; 6 Center for Systems Biology, Massachusetts General Hospital and Harvard Medical School, Boston, MA USA; 7 Ascension St. Vincent’s Birmingham, Birmingham, AL USA; 8 IBM Research, Yorktown Heights, NY USA; 9 Department of Pathology, Massachusetts General Hospital and Harvard Medical School, Boston, MA USA; 10 Department of Medicine, Massachusetts General Hospital and Harvard Medical School, Boston, MA USA; 11 Howard Hughes Medical Institute, Bethesda, MD USA

**Keywords:** Cancer genomics, Non-small-cell lung cancer, Tumour heterogeneity, Cancer

## Abstract

Although the development of multiple primary tumors in smokers with lung cancer can be attributed to carcinogen-induced field cancerization, the occurrence of multiple tumors at presentation in individuals with *EGFR*-mutant lung cancer who lack known environmental exposures remains unexplained. In the present study, we identified ten patients with early stage, resectable, non-small cell lung cancer who presented with multiple, anatomically distinct, *EGFR*-mutant tumors. We analyzed the phylogenetic relationships among multiple tumors from each patient using whole-exome sequencing (WES) and hypermutable poly(guanine) (poly(G)) repeat genotyping as orthogonal methods for lineage tracing. In four patients, developmental mosaicism, assessed by WES and poly(G) lineage tracing, indicates a common non-germline cell of origin. In two other patients, we identified germline *EGFR* variants, which confer moderately enhanced signaling when modeled in vitro. Thus, in addition to germline variants, developmental mosaicism defines a distinct mechanism of genetic predisposition to multiple *EGFR*-mutant primary tumors, with implications for their etiology and clinical management.

## Abstract (web summary)

Burr et al. analyze multiple primary *EGFR*-mutant lung tumors from ten patients and define developmental mosaicism as a mechanism underlying multiple tumor presentation in this setting.

## Main

As many as 10% of patients with non-small cell lung cancer (NSCLC) present with radiographic findings suggestive of two or more anatomically distinct synchronous lesions. This fraction is rising with the overall increased utilization of computed tomography (CT) imaging and specifically with improved utilization of low-dose CT screening in high-risk individuals with heavy smoking histories^1–4^. In such patients, multiple independent primary tumors are genetically unrelated, typically showing distinct genetic drivers, with mutational signatures of carcinogen-mediated DNA damage^1,5,6^. The concept of field cancerization in lung and other tissues, such as ultraviolet-exposed skin, explains the lifelong risk of multiple primary tumors and the need for regular cancer screening and monitoring in a subset of patients^1,5–13^.

NSCLC harboring activating mutations in the *EGFR* gene account for approximately 15% of all cases^14^. Specific, somatically acquired, ‘canonical’ mutations (most commonly L858R and delE746–A750) strongly activate receptor signaling, driving tumorigenesis and leading to dramatic clinical responses to epidermal growth factor receptor (EGFR) kinase inhibitors^15,16^. Tumors with canonical *EGFR* mutations typically do not show smoking-associated mutational signatures and the dramatic enrichment of cases among never smokers with NSCLC (up to 50% of cases) indicates that these mutations are linked to other risk factors^17,18^. Remarkably, the incidence of *EGFR*-mutant NSCLC is almost twice as high in women, compared with men, and in Asian populations, compared with non-Asian populations^19^. Although germline genetic polymorphisms linked to an increased prevalence of *EGFR*-mutant NSCLC have not been identified, shared haplotypes have been described between Asian and South American populations at risk of *EGFR*-mutant cancer^19–22^.

Canonical, somatic activating *EGFR* mutations have never been observed in the germline, suggesting that their cellular signaling activity is incompatible with normal embryonic development. However, we previously identified a family with inherited susceptibility to *EGFR*-mutant lung cancer, caused by a germline variant with attenuated signaling activity^23^: the T790M ‘gatekeeper’ mutation, commonly associated with acquired drug resistance to first- and second-generation EGFR inhibitors^24^. In this family, inheritance of a germline T790M-*EGFR* mutation confers weakly enhanced EGFR signaling, which may be tolerated during lung development. As multiple tumors emerge in susceptible family members, these show somatic acquisition of a canonical *EGFR* mutation in *cis* with the inherited variant, with the two mutations having synergy and enhanced activated signaling^25^. Although extraordinarily rare, familial susceptibility to NSCLC caused by an inherited *EGFR* T790M allele has since been confirmed in a few additional families^26,27^. Other rare *EGFR* germline familial variants, including V843I, R776X and P848L, have been reported^28^, with less confidence about their associated cancer risk.

In the absence of smoking-associated field cancerization or known familial predisposition, the presence of multiple synchronous *EGFR*-mutant tumors appears paradoxical and several distinct models have been proposed. Deep sequencing of normal lung tissues from individuals without cancer has revealed ultra-rare oncogenic *EGFR*-mutant alleles within as many as 18% of samples^29^, consistent with the emerging appreciation that cancer-causing mutations may populate apparently healthy aging tissues^30–34^. In that study^29^, pollution-associated inflammation is proposed as a potentially important modifying enhancer of spontaneous *EGFR*-mutation-driven tumorigenesis. Any tumors ultimately derived from such mutant *EGFR*-harboring cells would constitute independent, genetically unrelated events. On the other hand, previous studies of multiple primary *EGFR*-mutant lung cancers have indicated the presence of shared mutations, leading to the suggestion that they may be clonally related metastases, potentially resulting from intrapulmonary spread through lymphatics and possibly even air spaces, in the absence of disseminated metastatic disease^5,6,9–13^. However, such localized intrapulmonary mechanisms of dissemination do not readily explain involvement of different lobes and contralateral lungs, which may be observed in such cases. Moreover, many lung lesions in patients with *EGFR*-mutant multiple primary tumors are histologically preinvasive, without evidence of lymphovascular or visceral pleural invasion, thereby reducing the likelihood of metastatic spread. Given these divergent models, we applied genome-wide analytic strategies to test whether other genetic mechanisms may explain *EGFR*-mutant lung cancers presenting with multiple primary lesions.

## Results

### Patient clinical characteristics

**Fig. 1:**
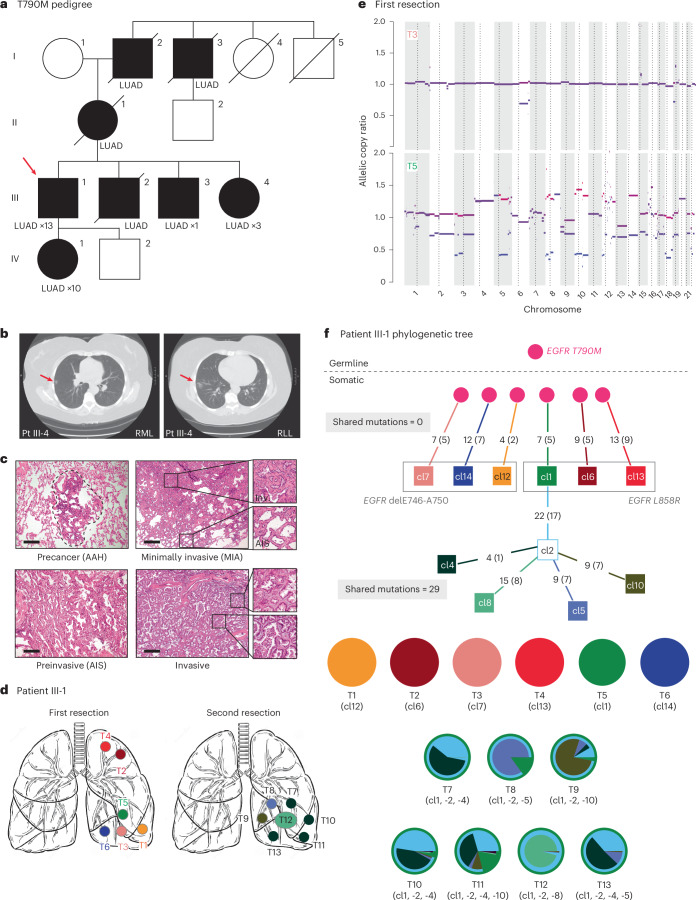
Genetic analysis of familial lung cancer caused by inherited T790M mutation in the *EGFR* gene. **a**, Pedigree of a family with multiple cases of lung adenocarcinoma, in which the index case (III-1) was diagnosed with six primary carcinomas (first resection), followed by resection of seven tumors 10 years later. Individuals shown in black have a confirmed or obligate germline T790M*-EGFR* mutation and those who have developed lung adenocarcinoma are denoted LUAD. The pedigree was minimally altered to preserve confidentiality (males, square; females, circles). **b**, CT scans of two tumors from patient III-4, one in the right middle lobe (RML) and the other in the right lower lobe (RLL). **c**, Histology of tumors from *T790M-EGFR* family patients showing the range of invasiveness encountered in our cohort, from precancerous AAH (patient III-4 lesion T2), to AIS (patient III-4 lesion T2), to MIA (patient III-1 lesion T2), to invasive adenocarcinoma (patient III-1; lesion T12). Panels are at ×40 magnification, with insets at ×200. Scale bars, 1 mm. **d**, Schematic of the tumor locations in patient III-1, at the first resection (left) and the second resection 10 years later (right). **e**, Copy number data for two tumors from the first resection of patient III-1. **f**, Phylogenetic lineage tracing of multiple tumors from patient III-1 based on WES. The tumors from the first resection, T1–T6, share no mutations outside of *EGFR*, as represented on the tree by no intersection point for clones 1, 6, 7, 12, 13 and 14 and, in the pie charts, by no colors shared between them. In contrast, the tumors from the second resection, T7–T13, share 29 mutations, as represented by the long trunk leading from cl1 to cl2 before branching into cl4, -5, -8 and -10. In addition, the pie charts for these tumors are complex mixtures of these four clones and clones are shared among multiple tumors. Numbers on branches are mutations that accumulated between two nodes, which represent distinct clones identified by WES. Numbers in parentheses are exonic mutations that are not in the FFPE context.

**Extended Data Fig. 1:**
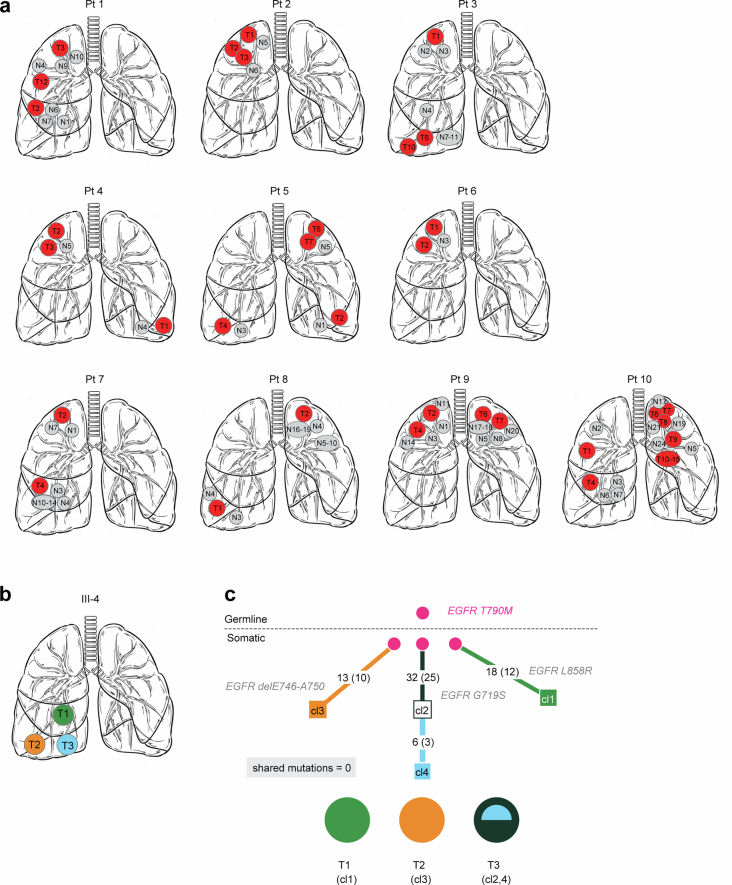
Sample locations and patient III-4 data. **a**, Schematics of the relative location of all normal samples and corresponding tumors in our cohort. **b**, Schematic of the tumor locations in patient III-4. **c**, Lineage tracing of patient III-4 with a known germline T790M-EGFR mutation, identified by Whole Exome Sequencing.

We identified ten patients from medical records at Massachusetts General Hospital (MGH) who had surgery between 2004 and 2019 for multiple early stage, spatially distinct, lung adenocarcinomas, with at least one specimen positive for the *EGFR* mutation by routine clinical genotyping (Table 1, patients 1–10). No patient had received any treatment before surgery and none was found to have lymph node involvement or suspected metastatic disease. Four patients were never smokers, three had a remote smoking history of <5 pack-years and three had a former >30-pack-year smoking history. None of the ten patients had a family history that was considered remarkable for multiple malignancies, including lung cancer. Tumor diameters ranged from 0.3 cm to 3 cm. In five patients, multiple tumors involved bilateral lungs, whereas, in three patients, tumors arose within different lobes on the ipsilateral side and, in two, they were confined to a single lobe (Table 1, Supplementary Table 1 and Extended Data Fig. 1a). Histologically, they were classified as precancerous atypical adenomatous hyperplasia (AAH, two tumors from two cases), adenocarcinoma in situ (AIS, one case), minimally invasive adenocarcinoma (MIA, ten tumors from six cases), mixed AIS and MIA (one case) and invasive adenocarcinoma (seventeen tumors from eight cases). In addition to the above cases of sporadic lung cancer, we applied our molecular analyses to a family with known germline transmission of an *EGFR* T790M allele (noncritical clinical features in the family history have been changed to preserve confidentiality) (Table 1, Fig. 1a and Extended Data Fig. 1b)^23^. In this family, the number of tumors per individual mutation carrier ranged from 1 to 13, with histology ranging from AIS to invasive adenocarcinoma (Fig. 1b,c and Supplementary Table 1). We first validated our tumor molecular analyses in one patient from this family with known germline susceptibility and then applied the same analytics to the sporadic cases with multiple primary tumors.

**Table 1:** Demographic and clinical characteristics of the patients

Patient	Age decade (gender)^a^	Smoking	Genetic ancestry	Stage	No. of tumors	Contralateral/Multilobar	Classification
III-1	50s (M)	Moderate	European	0	>13	Y/Y	Germline
III-4	60s (F)	Never	European	0	10	Y/Y	Germline
1	60s (F)	Former	European	IA	4	N/Y	Independent^c^
2	80s (M)	Former	European	IA	3^b^	N/N	Metastatic
3	50s (F)	Never	African	IA	>3	N/Y	Metastatic
4	50s (F)	Former	Asian	IA	>5	Y/Y	Germline
5	50s (F)	Never	African	IA	5	Y/Y	Germline
6	70s (F)	Former	European	IIB	4	N/N	Germline^c^
7	70s (F)	Never	European	IB	2	N/Y	Mosaic
8	80s (F)	Former	European	IA	4	Y/Y	Mosaic
9	60s (F)	Never	European	I	4	Y/Y	Mosaic
10	70s (F)	Former	European	IA	4	Y/Y	Mosaic

^a^Self-reported gender.

^b^Additional tumors observed on pathological assessment.

^c^Solely inferred from WES.

Clinical characteristics of two patients from a familial lung cancer pedigree (III-1 and III-4) and ten patients with sporadic multiple primary lung cancers. The stage refers to the clinical impression at the time of surgical resection following the International Association of Small Cell Lung Cancer, 8th edn staging guidelines. The number of tumors corresponds to those observed on CT scans throughout the patient’s lifetime. F, Female; M, Male; N, No; Y, Yes.

### Molecular evolution in familial *EGFR*-mutant lung cancer

Multiple specimens were available for two patients in the family with an inherited T790M mutant *EGFR* allele. For the index case (patient III-1), six geographically distinct lesions were resected from the left upper and lower lobes at the time of initial diagnosis, and an additional seven lesions resected 10 years later from the left lower lobe (Fig. 1d). WES of macrodissected, formalin-fixed paraffin-embedded (FFPE) tumor sections, compared with normal blood specimens, confirmed the heterozygous T790M-*EGFR* germline mutation in all tissues. DNA copy number alterations (CNAs) for two representative independent tumors from the first resection are shown in Fig. 1e, including T5, which subsequently gave rise to metastatic disease. Tumor T3 is an MIA and shows an allelic copy ratio of 1.0 for most chromosomes, indicating diploidy. Tumor T5 is an invasive adenocarcinoma and shows extensive aneuploidy. All tumors shared the functionally attenuated T790M germline mutation and showed subsequent somatic acquisition of a single secondary canonical *EGFR* mutation, either L858R or an exon 19 deletion mutation. However, beyond *EGFR*, the initially resected six tumors have no shared somatic mutations, consistent with their independent origin in the setting of cancer predisposition (Fig. 1f and Supplementary Table 2). A summary of cluster assignments is available in Supplementary Table 3.

In contrast, exome sequencing and phylogenetic reconstruction of the seven tumors resected from this patient 10 years later (T7–T13) show that they share between 29 and 33 somatic mutations (average 18.4% of all mutations are shared between the initial lesion T5 and the later lesions T7–13, and average 76.1% of all mutations shared across the later lesions T7–13) (Fig. 1f and Supplementary Table 4). Published analyses of intratumoral heterogeneity in lung adenocarcinomas indicate that any two regions of the same tumor share approximately 70% (interquartile range (IQR) 50–80%) of all mutations detected by WES^17^. Using this as a benchmark for our analysis, we conclude that the later tumors were recurrent metastatic foci, derived from one of the originally resected tumors (T5). This proof-of-concept analysis illustrates the genetic parameters that define completely independent early lung tumors versus metastatic recurrences from a single primary tumor, all arising within the context of an inherited genetic susceptibility.

### Developmental mosaicism in multifocal lung cancer

**Fig. 2:**
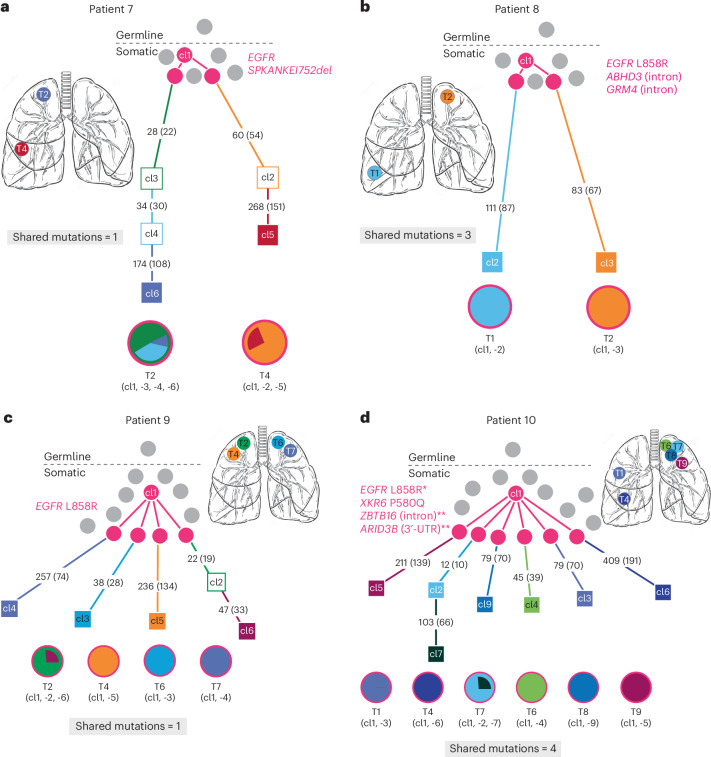
Mosaic somatic *EGFR* mutations mediate multiple primary lung tumors resulting from shared common ancestors. WES-derived phylogenetic trees of four cases with multiple tumors that share a common somatic ancestor. The shared somatic mutations, including *EGFR*, are shown in magenta. Numbers on branches are mutations that accumulated between two nodes, which represent distinct clones identified by WES. Numbers in parentheses are exonic mutations that are not in the FFPE context. In comparison with the previous cases, the branches of these trees do intersect at the pink clones, indicating some shared genetic ancestry that is not observed in completely independent or germline tumors. However, the number of shared mutations and the trunk of shared ancestry are very small relative to the total number of mutations in each clone. This is distinct from the patients with metastatic cancer. The pie charts of these tumors all exhibit the pink clone but are otherwise relatively simple and do not share clones between tumors. **a**, In case 7, the two geographically distinct tumors share an extremely rare somatic *EGFR* mutation, SPKANTKEI752del, and then acquire 236 and 328 separate exonic mutations. **b**, In case 8, two geographically distinct contralateral tumors share the recurrent mutation L858R, in addition to two somatic mutations, before acquiring 111 and 83 separate mutations each. **c**, In case 9, two tumors (T2 and T6) share the L858R mutation before acquiring between 38 and 69 private mutations. T4 and T7 were too early stage and of too low purity to assess by WES whether they also carry the mutation; however, clinical sequencing confirmed the L858R mutation in both. **d**, In case 10, six tumors share XKR6 P580Q, ZBTB16 (intron) and ARID3B (3′-UTR) mutations. ^*^EGFR L858R was found in three of the six tumors (T1, T6 and T7) by WES and a fourth (T4) by clinical genotyping. ^**^ZBTB16 and AIRD3B mutations were observed in multiple tumors but not normal tissue samples, therefore they are borderline for FFPE filtering. The other two tumors are early stage and low purity. These tumors went on to acquire 45–409 private mutations.

Having used our molecular analysis to distinguish independent primary tumors from metastatic recurrences in the setting of familial *EGFR*-mutant lung cancer, we turned to a separate cohort of ten apparently sporadic cases with multiple *EGFR*-mutant lesions. Four of these cases (patients 7–10) were unusual in that WES revealed shared somatically acquired mutations across anatomically separate tumors that were not observed in normal tissues (range 1–4 shared mutations, representing 0.4–4.0% (average 1.6%) of all exonic mutations) (Fig. 2 and Supplementary Tables 2–5). This pattern, with a small number of shared mutations across anatomically distant tumors, is incompatible with completely independent tumors arising either spontaneously, for which we would expect no shared mutations, or in the context of germline genetic predisposition, for which we would expect no shared mutations other than the germline *EGFR* mutation itself. It is also readily distinguishable from clonally related metastatic lesions, which would have a much higher fraction of shared somatic mutations, as demonstrated for patient III-1.

**Extended Data Fig. 2:**
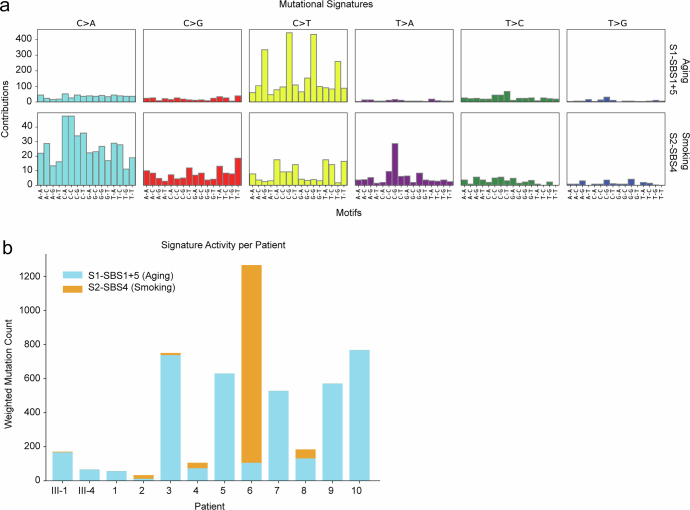
Mutation signature analysis of WES data. **a**, The mutational spectrum across all patients in this cohort is denoted, with each color representing one of the six potential base substitutions, and each substitution further stratified based on the flanking nucleotides. These spectra were decomposed into distinct signatures using a Bayesian NMF approach (SignatureAnalyzer) and compared to a database of known signatures (COSMIC). SignatureAnalyzer discovered signatures for aging (SBS1 and SBS5) and smoking (SBS4, cosine similarity=0.93). There is a small contribution of APOBEC (SBS2 and SBS13) in S2. **b**, Adding up all the probabilities associated with a particular signature (across all mutations), yields the expected mutational burden for that signature. The sum of these signature-specific mutational burdens (across all signatures) is the total number of mutations found in a given sample. The bar chart represents the mutational burden associated with each signature per sample.

Given FFPE as the source of tumor tissue, we separately scored mutations according to tissue context, excluding those whose presence in more than two different cases might indicate a mutation caused by formalin damage. Even with rigorous correction for this possible artifact, the evidence for a shared somatic origin for multiple tumors in cases 7–10 is retained. Mutational signature analysis of these tumors revealed expected smoking, APOBEC and aging signatures consistent with patient clinical characteristics and did not identify any unusual patterns (Extended Data Fig. 2 and Supplementary Table 6). Individual tumors within these cases also developed common lung adenocarcinoma driver events, such as *KRAS* and *TP53* mutations, in addition to the *EGFR* mutations, but these mutations were private to individual tumors (Supplementary Table 2). In contrast, *EGFR* mutations themselves were identical across anatomically distinct tumors within individual patients: in one case (patient 7), geographically distinct tumors shared a very rare mutation (SPKANKEI752del) that is unlikely to have developed twice independently and, in another case (patient 8), two tumors shared two unique somatic mutations, in addition to the common canonical *EGFR* L858R mutation (Fig. 2a,b and Supplementary Table 2). These data suggest developmental mosaicism: distant shared somatic ancestry among multiple primary tumors within individual patients as a result of early acquisition of an oncogenic mutation in a single cell with mutated daughters that are distributed throughout the adult body during the normal course of development. In cases where the developmental mosaicism is of oncogenic *EGFR* mutations, as shown here, developmental mosaicism lays the foundation for genetic predisposition to NSCLC.

**Fig. 3:**
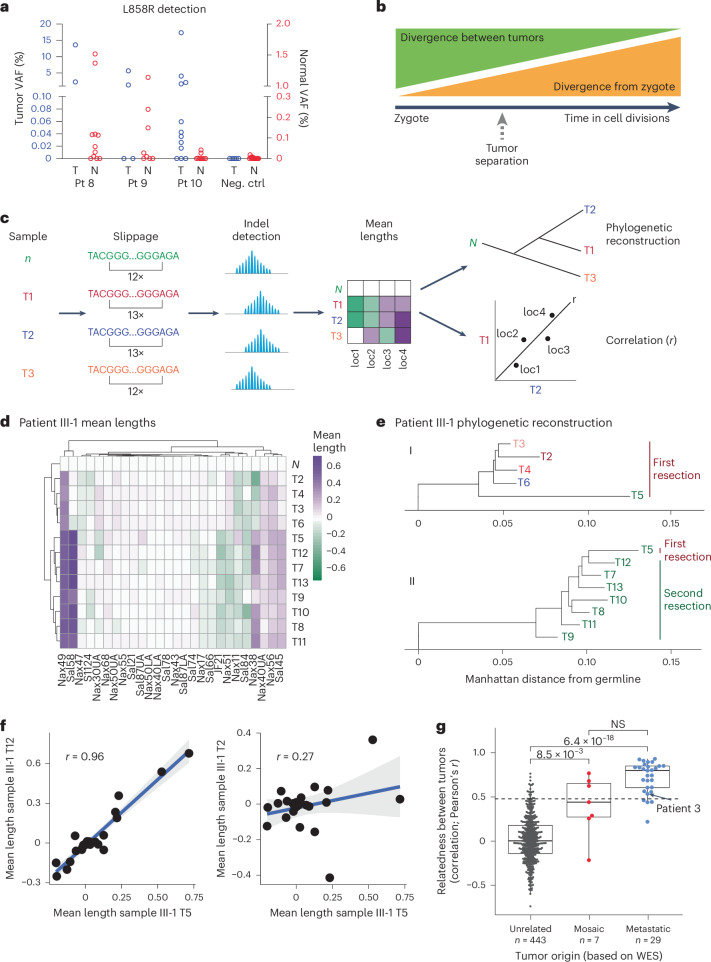
Developmental mosaicism is demonstrated by mutated normal lung cells and early common ancestors. **a**, Detection of the L858R *EGFR* mutation using ddPCR in cases where the tumor harbors the L858R mutation (patients (Pt) 8–10) compared with cases where the tumor contains another *EGFR* mutation (negative control (Neg. ctrl), patients 1 and 7). The VAF for tumors is on the left *y* axis whereas the lower VAF in normal samples is on the right *y* axis (*n* = 2 tumor and 11 normal samples (independent microdissection regions) for patient 8; 4 tumor and 8 normal samples for patient 9; 12 tumor and 9 normal samples for patient 10; 3 tumor and 6 normal samples for patient 1; and 2 tumor and 9 normal samples for patient 7). **b**, A schematic showing the relationship between divergence from zygote and divergence between tumors. **c**, Schematic of poly(G) genotype analysis method, based on ref. ^38^. Samples collected from a single patient have poly(G) sites that may have undergone slippage due to hypermutability. The assay detects these indels and measures their mean length change compared with normal tissue. The correlation between the two tumors across all poly(G) sites is represented by Pearson’s correlation coefficient (*r*). **d**, Heatmap showing the mean distance from normal lung for each poly(G) hypermutable region, for each tumor from patient III-1. Tumors that have similar patterns are more closely related than tumors that have different patterns. **e**, Phylogenetic tree of patient III-1 based on the poly(G) analysis. The tree is rooted at the germline sample: i shows only samples from the first resection and ii samples from the second resection plus recurring sample T5. **f**, Correlation plots between tumors from patient III-1. The dots represent the mean length from normal at each poly(G) location (*n* = 26 poly(G) loci). The *r* estimates what fraction of cell divisions were shared between the tumor pair before divergence. The gray shading represents the 95% CI. **g**, Poly(G) relatedness between tumor pairs. Each point represents the poly(G) evolutionary distance between two tumors from cases that are unrelated (different individuals), mosaic or metastatic (classification based on WES analysis of exonic mutations). The dotted line represents that correlation coefficient >95% of the unrelated tumor pairs. Boxplot elements: center line, median; box limits, lower and upper quartiles; whiskers, lowest and highest value within 1.5× the IQR. *P* values are a Holm–Bonferroni-corrected, post-hoc Dunn’s test after a significant Kruskal–Wallis test. The arrow is the tumor pair from metastatic patient 3. NS, Not significant. (*n* = 443 interpatient tumor pair comparisons across eight patients for the unrelated analysis; *n* = 7 intrapatient tumor pair comparisons within four mosaic patients for the mosaic analysis; and *n* = 29 intrapatient tumor pair comparisons within two metastatic patients for the metastatic analysis). Source data

Developmental mosaicism in adult tissues predicts that variant alleles may be present, albeit at very low frequency, within normal tissues, where they may comprise a reservoir of cells susceptible to transformation^35^. To detect such cells, we employed droplet digital PCR (ddPCR) technology, which counts individual DNA molecules with a detection limit of ≤0.01% of cells within a population^36^. In all three putative mosaic cases with L858R *EGFR*-mutant tumors, the L858R mutation was detectable in multiple, anatomically distinct, normal lung samples, albeit at much lower allele fractions than in the tumor samples (Fig. 3a). As a control, no such mutations were detected within normal tissues of cases with *EGFR* mutations other than L858R (patients 1 and 7). Although we cannot formally exclude clonal hematopoiesis as a source of rare mutant alleles within normal tissues, we note that canonical *EGFR* mutations are not among the known recurrent events reported in clonal hematopoiesis and the *EGFR* mutations were observed only in cases with matched tumor harboring the same mutation. Thus, molecular analyses suggest the presence of very rare cells harboring shared *EGFR* mutations within normal lung tissues of patients with potentially mosaic-derived *EGFR*-mutant cancers.

To quantify the divergence between tumors, we generated poly(G) fingerprints^37–39^, which measure insertions/deletions (indels) in hypermutable guanine mononucleotide repeats. These mutations occur at high rates during DNA replication as a consequence of polymerase slippage^40^ (Fig. 3b,c). Therefore, the divergence between the poly(G) genotypes of two somatic cell populations is a reflection of the number of cell divisions that separate them. We first benchmarked the poly(G) assay on samples from *EGFR* T790M familial cancer patient III-1. Poly(G) analysis demonstrated a short shared evolutionary history and few shared variants among the tumors from the first resection, consistent with the results from the WES analysis. In contrast, the metastatic lesions from the second resection 10 years later showed a long shared trajectory and close genetic concordance (Fig. 3d,e). In accordance with the WES results, poly(G) analysis conclusively showed that tumor T5 from the original resection was the source of metastatic disease.

To enable a quantitative comparison of the genetic relatedness between any pair of tumors from the same patient, we calculated Pearson’s correlation coefficients among the tumors’ poly(G) genotypes (Supplementary Table 7). The correlation coefficient estimates the fraction of cell divisions in the history of two tumors that they have spent as part of the same lineage^41^. Crucially, this estimation does not depend on knowledge of the underlying mutation rate or purity of the tumors, thereby providing an unbiased view of their evolutionary history. Thus, tumor samples that share a large fraction of their evolutionary history (such as metastases) show high correlations (Fig. 3f, left panel T5 and T12), whereas tumors that share limited evolutionary history display low correlations (Fig. 3f, right panel T5 and T2). To determine the evolutionary history that two unrelated tumors would be expected to share by chance, we calculated the correlation between all tumor pairs from different patients (Fig. 3g, unrelated group). Indeed, the average correlation between known unrelated tumors was 0.02 (95th percentile = 0.46) (Fig. 3g, dotted line). The genetically related tumors from patient 3, identified as metastatic by abundant shared exonic mutations, displayed a correlation of 0.53 (>95th percentile for unrelated tumors) (Fig. 3g, arrow). These tumors thus underwent an expected fraction of 53% of their cell divisions as part of the same lineage, consistent with the large number of shared exonic mutations.

Poly(G) analysis of patients 7–10, with potential mosaically derived, multiple primary tumors, supports the notion that the lineages giving rise to these cancers diverged relatively early in time (Supplementary Table 7). On average, the tumors in these patients are less closely related than bona fide metastases, but they share a longer developmental history than completely independent tumors. We calculate that the lineages giving rise to these tumors underwent, on average, 44% of their cell divisions together before separating, compared with 80% for metastases and 0% for unrelated tumors (Fig. 3g).

Remarkably, three of the four patients with potential mosaically derived primaries had tumors located in contralateral lungs and one had tumors located in two different lobes of the same lung (Extended Data Fig. 1a), yet histopathological analysis in all 4 patients shows only 4 of 14 tumors to be invasive adenocarcinomas, with the other 9 lesions being minimally invasive (Supplementary Table 1). Thus, both genetic data and histopathology suggest that metastasis is unlikely to explain the appearance of multiple primary tumors in these cases. Consistent with the sequencing and poly(G) lineage studies, the fact that distinct tumors commonly arose in contralateral lungs suggests that their divergence may have occurred early in lung development, with their progeny seeding disparate regions of the adult organ. Taken together, these analyses point to shared ancestry and early divergence, consistent with developmental mosaicism.

### Germline *EGFR* variants in multifocal lung cancer

**Fig. 4:**
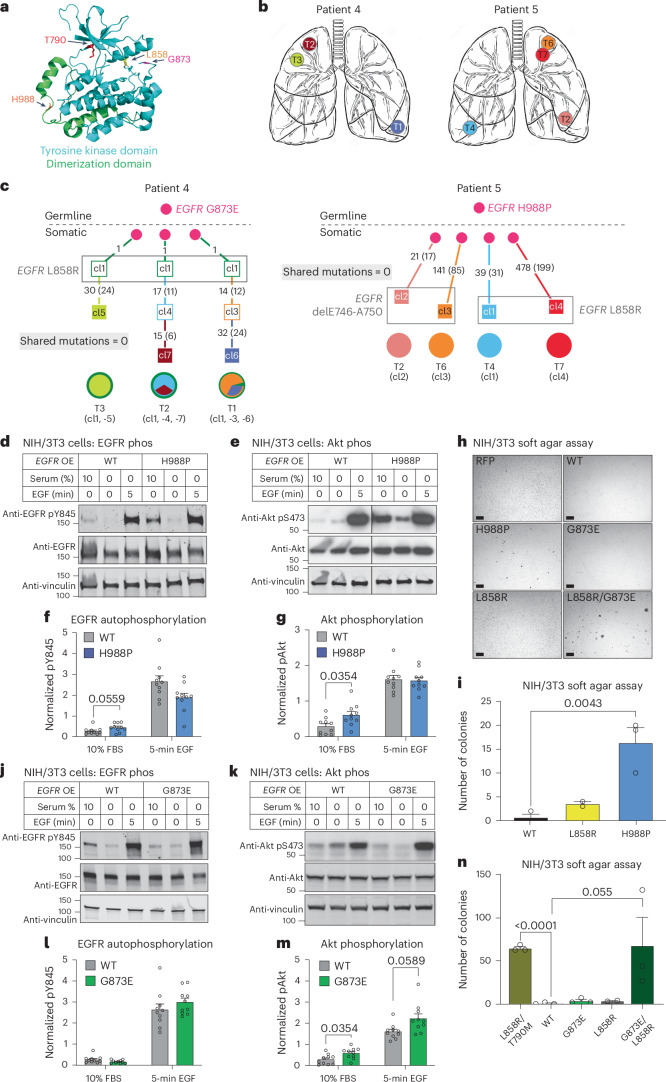
Germline H988P and G873E mutations increase EGFR activity. **a**, Position of the relevant residues within a partial EGFR protein crystal structure (aligned PDB structures EGFR 696-1022 T790M (5gty) and EGFR 703-985 (4zjv)). The dimerization domain is green and the catalytic tyrosine kinase domain blue. **b**, Schematic of tumor locations in patients 4 and 5. **c**, Lineage tracing of patient 4 and 5 tumors, derived from WES. Numbers on branches are mutations that accumulated between two nodes, which represent distinct clones identified by WES. Numbers in parentheses are exonic mutations that are not in the FFPE context. **d**–**g**,**j**–**m**, Functional effect of the H988P-*EGFR* mutant (**d**–**g**) or G873E mutant (**j**–**m**), compared with the WT construct. **d**,**e**,**j** and **k** are western blots and **f**,**g**,**l** and **m** are quantifications of the blots immediately above. The pY845 was normalized to vinculin, then total EGFR and finally to the average signal within an experiment for comparison across experiments. phos, phosphorylation. OE, overexpression. EGFR and pY845 are both rabbit antibodies and were run on different blots (processed in parallel), each with their own vinculin loading control, which was used for quantification. A representative vinculin blot is shown in the figure. The pS473 was normalized to a vinculin sample processing control from a matched EGFR blot, then to the average signal within an experiment. AKT and pS473 are both rabbit antibodies and were run on different blots (processed in parallel). Images vertically sliced to juxtapose nonadjacent lanes were run on the same gel (*n* = 10 biologically independent samples per figure). Data are presented as mean values ± s.e.m. A two-way ANOVA was performed to determine statistical significance and false recovery rate (FDR) *q*-values were corrected for multiple comparisons using the Benjamini, Krieger and Yekutieli procedure. **h**,**i**,**n**, Representative images of colony formation by NIH/3T3 cells in soft agar in cells expressing WT or mutant *EGFR* constructs (**h**). Scale bars, 100 μm. This experiment was repeated 3× with similar results, as quantified in **i** and **n**. Quantification data of colonies at least 20 μm in size is presented as mean values ± s.e.m. *P* values are a one-tailed, unpaired Student’s *t*-test not corrected for multiple comparisons. Source data

**Extended Data Fig. 3:**
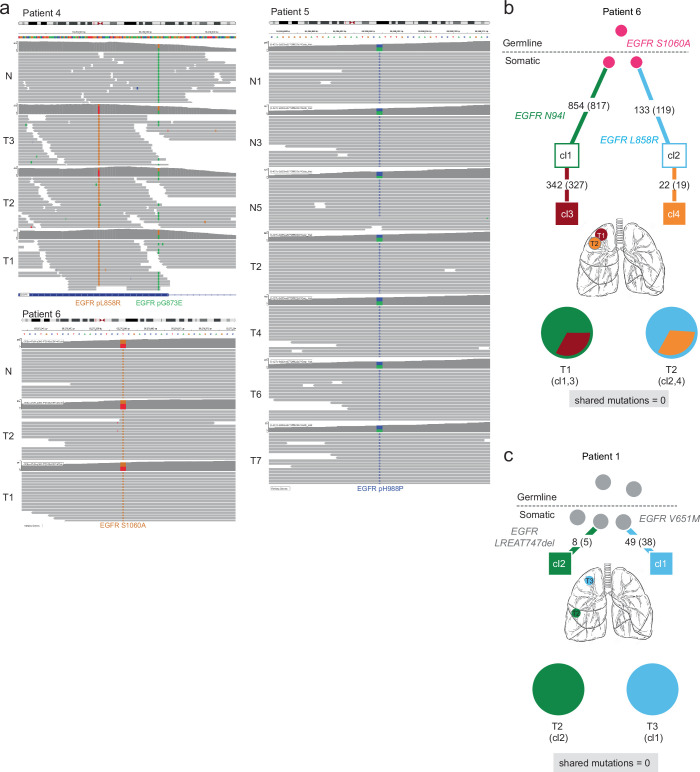
Germline mutations and patient 1 and 6 WES results. **a**, IGV tracks for the patients harboring germline variants, showing the germline EGFR mutations in the normal samples as well as tumors. Patient 4 also shows EGFR G873E on same read as L858R in the tumor samples, indicating that the two mutations are in cis. **b**, Phylogenetic tree from patient 6, with a germline variant identified by WES. **c**, Phylogenetic tree from patient 1 predicted to be independent by WES.

In two other cases with apparently sporadic multiple primary *EGFR*-mutant tumors, we identified uncommon heterozygous germline *EGFR* variants in normal lung tissue. The lung tumors showed the same heterozygous mutation, along with a second, somatically acquired canonical *EGFR* mutation. In these two cases, the germline *EGFR* variants are within critical functional domains of the protein: patient 4 has a G873E mutation within the tyrosine kinase domain (exon 21) and patient 5 has a H988P mutation within the dimerization domain of EGFR (exon 25) (Fig. 4a). Both of these patients had bilateral synchronous lesions at presentation (Fig. 4b). WES identified no shared somatic mutations outside of the *EGFR* gene, indicating independent primary tumors arising in the setting of potential genetic predisposition, analogous to the first six tumors characterized in index patient III-1 from the prototype T790M-*EGFR* family (Supplementary Table 2 and Fig. 4c). When present in the germline, T790M-*EGFR* is a weakly activating allele and lung cancers show somatic acquisition of a canonical *EGFR* activating mutation in *cis* with the germline allele, resulting in a strongly activating protein with both mutated residues^23,25^. In patient 4, the secondary canonical mutation L858R was also found in *cis* with the germline allele in all three tumors; patient 5 also had secondary canonical mutations (L858R, exon 19 delE746–A750, R108G), but, given the position of the relative mutations, the length of sequencing reads precluded determination of whether the heterozygous secondary mutation arose in *cis* or in *trans* with the heterozygous germline allele (Extended Data Fig. 3a and Supplementary Tables 1 and 2).

The *EGFR* mutation H988P has been previously identified as a variant of unknown significance^42^. Of note, patient 5 had African ancestry and this variant appears to be relatively common in the African ancestry population (2.4% prevalence), compared with the white population (<0.1%)^43^. To further explore the population effects of this variant, we examined the allele frequency in TCGA tumors from patients with African ancestry. We observed a two- to threefold higher prevalence of the germline variant in cancer compared with the frequency reported in gnomAD (general population excluding cancer patients). In addition, many of these patients had *EGFR* amplifications, even in cancers that are not typically driven by *EGFR*. The existing detailed molecular analyses of *EGFR*-mutant lung cancers in the African population are insufficient to provide insight as to potentially increased cancer risk that may be associated with this allele, which is classified as ‘likely benign’ in ClinVar^42^. We undertook functional reconstruction experiments of H988P-*EGFR* using standard signaling assays in mouse NIH/3T3 cells which do not express endogenous protein. Compared with wild-type (WT) EGFR, the H988P mutant shows modestly increased phosphorylation under unstimulated conditions, a measure of baseline receptor signaling activity, together with enhanced downstream signaling of its key mediator AKT serine/threonine kinase (Fig. 4d–g). H988P*-EGFR*-transfected NIH/3T3 cells also generate more colonies in soft agar, a prototype cell transformation assay (Fig. 4h,i). The second germline *EGFR* variant, G873E, has been reported as a somatic mutation and shown to play a role in resistance to gefitinib^36,44–46^. By itself, we find that it has modest activating capacity, but it is synergistic when combined in *cis* with the canonical L858R mutation (Fig. 4h,j–n). Importantly, all tumors from patient 4 had an L858R mutation in *cis* with the germline G873E mutation (Extended Data Fig. 3a). Thus, like the established familial T790M mutation, both H988P and G873E appear to have an attenuated proliferative effect that may be tolerated in the germline without compromising normal embryonic development. However, determining any clinically important cancer risk associated with these inherited alleles will require epidemiological studies.

Two other cases arising in minimal smokers harbored tumors that were genetically independent. In one case, patient 6, we identified a germline *EGFR* S1060A mutation, residing within the alternative splicing variant *EGFR-vA*, which is expressed at low levels in normal tissues (Extended Data Fig. 3b). The *EGFR-vA* isoform has been reported as potentially oncogenic in gliomas^47^, but we were unable to confirm aberrant EGFR signaling associated with S1060A, making it a variant of unknown significance. In the other case, patient 1, we did not identify a candidate functional germline mutation that might explain the independent somatic genetic origin of these tumors (Extended Data Fig. 3c).

**Fig. 5:**
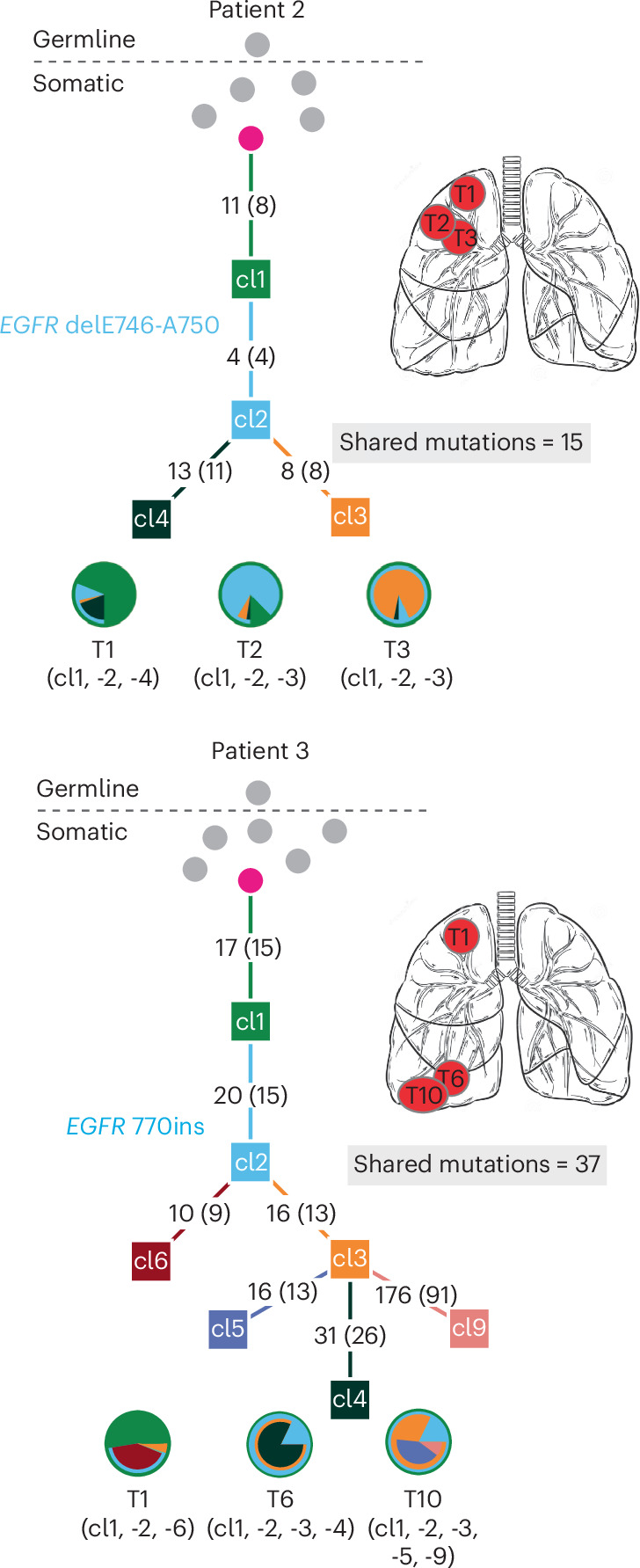
Lineage tracing of metastatic cancers. Phylogenetic trees from WES of two patients with metastatic cancer. The shared *EGFR* mutation is annotated on the tree. Numbers on branches are mutations that accumulated between two nodes. Numbers in parentheses are exonic mutations that are not in the FFPE context. These trees show a long trunk of shared mutations before branching into separate clones. Consistently, the pie charts are made up of mixtures of clones that are shared between tumors.

In the remaining two cases with multiple *EGFR*-mutant tumors (patients 2 and 3), molecular analysis indicated a metastatic relationship between the individual lesions. In both patients, recurrent disease was considered among the clinical possibilities at the time of resection. In these cases, WES and phylogenetic reconstruction showed that many mutations were shared across all tumors (16 and 37 shared mutations in patients 2 and 3, respectively, representing an average of 53.7% of all exonic mutations within a tumor (range from 46.0% to 61.3% per tumor)) (Fig. 5 and Supplementary Tables 2 and 4). This is consistent with the published range of 50–80% clonal mutations between two samples of the same tumor and with our findings in the T790M family^17^. Both cases subsequently had clinically recurrent cancer within 3 years of surgery.

## Discussion

**Fig. 6:**
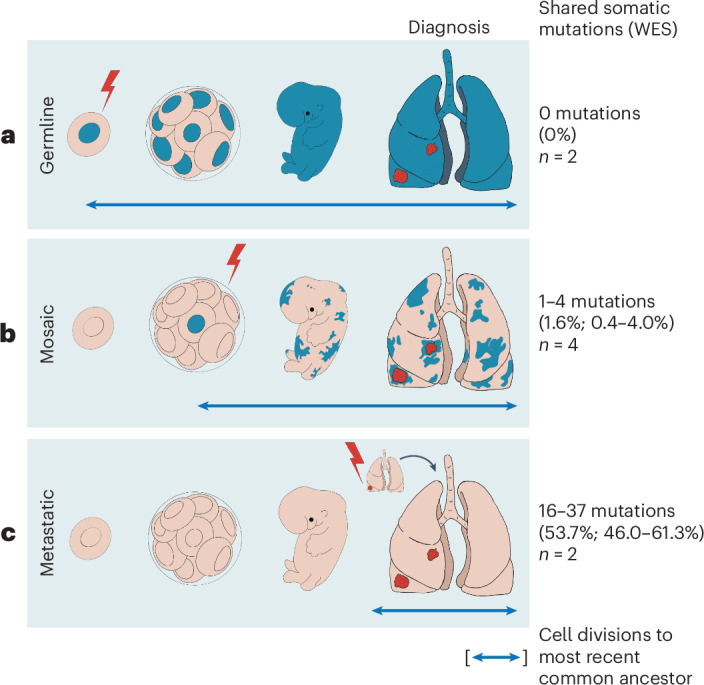
Genetic distinctions between multiple lung cancers with inherited, mosaic and metastatic origin. Schematic representation of three distinct mechanisms underlying multiple *EGFR*-mutant lung tumors. The average percentage of somatic mutations shared among different tumors is shown on the right (range in parentheses). **a**, In cases with inheritance of an attenuated mutant *EGFR* allele (either familial or apparent sporadic), the mutation presenting in the germline (lightning bolt) is shared by all somatic tissues. A second canonical *EGFR* mutation arises somatically at high frequency in predisposed lung cells, leading to multiple tumors with no shared somatic mutations. The evolutionary distance to the most common shared ancestor between different tumors (arrow) extends to the germline; 0 nongermline mutations are shared between tumors. **b**, Cases with mosaic predisposition arising from acquisition of an *EGFR* mutation during development (lightning bolt). This timing determines the proportion of normal cells containing the variant allele and the likelihood of developing multiple tumors. In addition to the activating *EGFR* mutation, a small number of additional somatic genetic variants are shared before the mosaic tissues diverge and acquire independent tumor-associated mutations. **c**, In sporadic cancers without genetic susceptibility, a single *EGFR* mutation arises in a somatic lung epithelial cell (lightning bolt), generating a single tumor. Metastases from this tumor share extensive mutational profiles and the most recent common ancestor for these multiple tumors is the primary tumor. *n*, number of analyzed cases in cohort.

We have shown that multiple primary *EGFR*-mutant lung cancers may arise from canonical activating *EGFR* mutations that are acquired early in development, leading to mosaicism for this driver mutation in the adult lung (Fig. 6). The multiple primary tumors that emerge in this setting share identical somatic canonical *EGFR* mutations along with mutations in other genes (0.4–4.0% of all exonic mutations), confirming their shared clonal origin. Poly(G) lineage analysis indicates that such mosaically derived tumors display common ancestry that is intermediate between the short shared lineage seen in independent tumors with germline predisposition and the longer shared lineage displayed by metastatic tumors. Other established causes of multiple primary *EGFR*-mutant tumors include an attenuated germline variant in the *EGFR* gene, as first demonstrated for the T790M allele^23^. The multiple primary tumors arising in the setting of germline predisposition share no somatic mutations, other than primary germline and secondary somatic *EGFR* mutations, and poly(G) analysis shows a limited shared evolutionary history among them. However, the possible lung cancer risk attributable to the candidate variants described in the present study needs to be determined using epidemiological studies. Such studies have limited power for rare germline variants, but the H988P allele has a relatively high prevalence in the African population, making it all the more important, given its possible clinical implication. Epidemiological analyses of germline H988P allele prevalence among *EGFR*-mutant lung cancers in the African American population should be undertaken, and the allele should be considered as having uncertain clinical relevance until then. Finally, metastatic lesions are also readily distinguished from mosaically derived primary tumors by their much higher fraction of shared mutations among them and with their primary tumor of origin (46.0–61.3% of all exonic mutations). Thus, the appearance of multiple primary *EGFR*-mutant tumors is biologically distinct from the metastatic spread of cancer and it may result from an initiating *EGFR* mutation, either in the germline or during early development.

The presence of multiple distinct primary *EGFR*-mutant cancers at the time of clinical presentation has long represented a conundrum. Two common hypotheses include intrapulmonary metastasis and field cancerization. Lung cancer metastasis characteristically presents with disseminated disease, but it may also arise through intrapulmonary lymphatic spread within adjacent regions of the lung. Local spread through air spaces has also been reported, particularly in some tumors with micropapillary and/or solid histology^48^. The detection of shared mutations between anatomically distinct primary lung tumors has made intrapulmonary spread the lead model to explain the presence of multiple primary tumors at presentation. Challenging this model of localized metastasis, however, is the fact that many lesions in patients with multiple primaries are histologically classified as preinvasive and even preneoplastic. Furthermore, their anatomical locations may include different lobes or contralateral lungs, which are probably beyond the reach of localized lymphatic or airspace spread. In the cases reported in the present study, we did observe two cases with metastatic disease, as suspected clinically and confirmed by their largely shared mutational composition. However, the four cases in which anatomically distinct primaries contain a number of shared somatic mutations comprising only a small fraction of the total exonic mutational burden are inconsistent with intrapulmonary metastasis.

The second common hypothesis, ‘field cancerization’, is a phenomenon whereby the entire tissue is damaged by carcinogenic exposure, leading to multiple independent and genetically unrelated tumors. It is most frequently applied to cases with a heavy smoking history, although exposures owing to environmental radon and occupational exposures have also been considered. Recent studies in barrier organs, including skin, esophagus and lung, have indicated that, as they age, histologically normal tissues may acquire canonically oncogenic mutations that give rise to small patches of clonal expansion, but without evidence of frank malignancy^30–34,49,50^. In the lung, deep sequencing reveals that up to 18% of normal samples have a detectable mutant *EGFR* allele and even more cases harbor *KRAS* and other mutated oncogenes^29^. The high prevalence of normal lung specimens with rare detectable *EGFR*-mutant alleles as a result of field cancerization does not explain the occurrence of the multiple *EGFR*-mutant primary tumors described in the present study, because they would be genetically independent, lacking the multiple shared mutations that define the developmental, mosaically derived primaries.

Our study hypothesizes that a canonical and fully activating *EGFR* mutation may arise early during lung development, creating a mosaic of lung epithelial cells harboring this mutation and distributed across the adult organ. Our analysis of poly(G) repeats in such tumors suggests that *EGFR* mutations may occur before cells have undergone half of the divisions on their way to tumor initiation. Although normal tissues other than lung were not available for our retrospective cohort, we were able to show the presence of a low frequency of *EGFR* mutations in normal lung tissues, only in the patients with multiple primaries whose tumors had the corresponding *EGFR* mutation. Unlike mutant *EGFR* alleles in the context of field cancerization, which represent random independent genetic events, these *EGFR* mutations within normal tissues may result from early developmental mosaicism, ultimately giving rise to anatomically disparate tumors that also share multiple passenger mutations, consistent with their common clonal origin. The mechanism by which *EGFR*-mutant lung epithelial cells generate premalignant and ultimately invasive cancers is unknown, but recent epidemiological and mouse modeling studies indicate that inflammation associated with air pollution enhances the likelihood that an *EGFR*-mutant cell will give rise to a malignant tumor^29^. In this context, the high frequency of mutant *EGFR* allele detection in normal lung is in marked contrast to their virtual absence (<0.1%) in normal skin^29^, raising the possibility that EGF signaling mediates distinct proliferative effects in the lung, thereby contributing to the persistence of mosaically derived progeny during early lung development.

The concept of mosaicism in human genetics is best illustrated by neurofibromatosis type 1 (NF1), where up to 5% of patients have segmental café-au-lait spots, attributable to a mutation in the *NF1* gene that arose during embryonic development and affects only a portion of the adult body^50,51^. In the brain, postzygotic mosaicism affecting Ras/Raf/MAPK signaling may play a role in the pathogenesis of mesial temporal lobe epilepsy^52^. In cancer, the concept of a clonally derived origin for multifocal sporadic cancers was first proposed based on X-chromosome inactivation studies in bladder cancer^53^. Mosaic-inactivating mutations have been reported in tumor-suppressor genes linked to high-risk cancer predisposition syndromes, including Li–Fraumeni and von Hippel–Lindau syndromes^54,55^. In the pediatric kidney cancer Wilms’ tumor, geographically distinct precursor lesions, called nephrogenic rests, share mutations with each other and with geographically distinct tumors, consistent with mutations arising during early renal organogenesis^35,56^. Although developmental mosaicism has not been reported as a common event in epithelial cancers affecting adults, one case report described the incidental discovery at autopsy of multiple precancerous lung lesions, all sharing an identical somatic *TP53* mutation, raising the possibility of a mosaic mechanism^57^. Estimating the population-level prevalence of developmental mosaicism in the lung will require follow-up studies using larger, multicenter, prospective patient cohorts.

The second genetic mechanism that we describe underlying multiple *EGFR*-mutant primary lung cancers extends the observation of rare familial lung cancer to cases without such pedigrees. As with familial transmission of a germline T790M-*EGFR* allele, the germline *EGFR* variants identified in the present study encode mutant proteins with modestly increased enzymatic activity, suggesting that, unlike strongly activating mutants, they are not deleterious during normal embryonic development. They may, however, be sufficient to increase the size of target cell populations within the lung, which undergo tumorigenesis after sustaining a second, more strongly activating *EGFR* mutation. To date, clinical sequencing efforts of *EGFR* in NSCLC have identified approximately 500 somatic variants of unknown significance, a small subset of which have been identified in the germline (ClinVar and gnomAD)^42,43,58^. Further studies will be required to determine how many additional *EGFR* variants have subtly enhanced signaling properties when present in the germline, potentially linked to increased tumorigenesis and their penetrance.

Finally, we note that understanding the etiology of multiple *EGFR*-mutant tumors with synchronous presentation may impact clinical treatment strategies, particularly for light/never smokers. Management of independent primary tumors includes lung parenchyma-sparing resections with curative intent. Mosaically derived tumors should not be confused with metastatic disease, despite the presence of some shared mutations. Moreover, when caused by either a germline *EGFR* variant or developmental mosaicism, patients presenting with multiple *EGFR*-mutant primary tumors are probably at risk of developing additional tumors during their lifetime, suggesting the importance of ongoing surveillance and raising consideration of new prophylaxis strategies.

## Methods

### Ethics statement

Two protocols were used for the present study, both reviewed and approved by the Dana Farber/Harvard Cancer Center (DF/HCC) Institutional Review Board, which oversees clinical cancer protocols for all Harvard institutions including MGH. For the tumor blocks from sporadic lung cancer cases, the approved protocol DF/HCC no. 13-416 includes permission for molecular analysis, privacy of results and publication of deidentified results. For the T790M familial pedigree, the cases were initially collected under protocol DF/HCC no. 94-138 at the time of the initial publication^23^ and reconsented under protocol DF/HCC no. 13-416 for the present study. Under both protocols, all participants provided written informed consent for collection of tumor and tissue specimens and clinical information for inclusion in a tissue repository for future research, including DNA and RNA sequencing, except where noted, and written informed consent for sharing of clinical information in deidentified publications.

### Patients

Ten patients were selected retrospectively from an institutional database as having undergone resection for two or more early stage lung cancers, with at least one lesion having a known *EGFR* mutation by next-generation sequencing analysis. These patients gave informed consent for their biological materials to be included in this database. These tumors were classified as early stage by the reviewing pathologist at the time, even though our genetic analysis suggests that two of the patients had metastatically related tumors. No patients had received systemic treatment for cancer preoperatively. Clinical histories were extensively reviewed (see Table 1 and Supplementary Table 1 for summary of patient and tumor characteristics). Patient genetic ancestry was inferred from electronic health records. Gender was self-reported and was not considered in the study design or analysis because we were too limited in our sample availability to stratify based on gender. An additional two patients with a known inherited *EGFR* T790M mutation were included for proof of concept.

### Specimen collection and histopathology

Sections from the entire tumor and representative lung parenchyma distinct from the tumor were fixed in formalin and embedded in paraffin. Histopathological slides were made from the FFPE sections and retrospectively reviewed by a single observer, expert pulmonary pathologist (M.M.K.), who histologically classified and staged individual tumors in accordance with the World Health Organization classification of lung tumors^59^ and the 8th edition American Joint Committee on Cancer lung cancer staging guidelines^60^, respectively. The areas of highest tumor purity and histologically normal lung tissue were also selected.

### TNA extraction protocol

Total nucleic acid (TNA) was extracted from designated tumor and normal lung tissue from each patient using the standard protocol of Agencourt Formapure kit (Supplementary Table 1). The approximate location of the normal samples relative to the tumors is shown in Extended Data Fig. 1a.

### Whole-exome sequencing

Before WES, a standardized PicoGreen dsDNA Quantitation Reagent test (Invitrogen) was used to quantify DNA in triplicate. The Fluidigm Genotyping fingerprint genotyping of 95 frequent SNPs was used for the quality control identification check (Fluidigm). Using the KAPA Library Prep kit and palindromic forked adapters from Integrated DNA Technologies, libraries were constructed from double-stranded (ds)DNA. Before hybridization, libraries were combined. Utilizing a 37-Mb target, hybridization and capture were carried out using the essential components of Illumina’s Rapid Capture Enrichment Kit. On the Agilent Bravo liquid handling system, the library building, hybridization and capture processes were all fully automated. Library pools were denatured on the Hamilton Starlet using 0.1 N NaOH after post-capture enrichment. DNA libraries were cluster amplified using HiSeq 4000 exclusion amplification reagents and HiSeq 4000 flowcells in accordance with the manufacturer’s (Illumina) instructions. HiSeq 4000 flowcells were sequenced using sequencing-by-synthesis chemistry. RTA (v.2.7.3) or later was then used to examine the flowcells. Sequencing of each pool of entire exome libraries was done using paired 76-cycle runs with two 8-cycle index reads across the number of lanes required to provide coverage for all libraries in the pool.

Sequence data were analyzed using the Broad Institute’s Cancer Genome Analysis WES Characterization Pipeline, in which aligned BAM files were inputted into a standardized WES, somatic, variant-calling pipeline as previously described^61^, which included MuTect (v.1.1.6) for calling somatic single nucleotide variants (sSNVs), Strelka2 (v.2.9.9) for calling small indels, deTiN (v.2.0.1) for estimating tumor-in-normal (TiN) contamination, ContEst (v.1.4-437-g6b8a9e1; GATK v.3.7.0) for estimating cross-patient contamination, AllelicCapSeg (v.22) for calling allelic copy number variants and ABSOLUTE (v.1.5) for estimating tumor purity, ploidy, cancer cell fractions and absolute allelic copy number. Artifactual variants were filtered out using a token panel-of-normals (PoN) filter, a blat filter, an OxoG filter and an FFPE filter.

### Filtering formalin fixation (FFPE) and other potential artifacts

FFPE and OxoG artifacts, which are developed with inherent lead strand asymmetry (orientation bias) owing to context specificity of the mutational processes, were filtered as previously described^61,62^. In brief, OxoG is an artifact signature resulting from oxidative damage to guanine during library preparation, which causes guanine to pair with adenine instead of cytosine, ultimately causing an observed G>T mutation. These artifacts will occur only on one strand whereas a somatic event will show the change on both strands of DNA, and this orientation bias is used to distinguish real events from artifacts. The cohort also had single nucleotide artifacts resulting from the use of FFPE samples, wherein formaldehyde causes deamination of cytosine resulting in C>T mutations similar to those of the aging signature, but with the same orientation bias observed in OxoG events, allowing us to use the same algorithm for determining orientation bias that has previously been used on FFPE samples^62^. Given the importance of this issue for mosaicism and tumor relatedness analysis, we have performed additional filtering, in which we have ‘force called’ the identified mutations across tumors and normals in the cohort. Force calling is the process of gathering the evidence for a mutation at a particular genomic site (that is, determining the number of mutated and WT-independent sequencing reads that cover the site). In standard WES depth, three or more mutated reads in the tumor and none in the normal are required to detect a somatic mutation at a non-noisy site. However, FFPE can dramatically increase the noise at particular sites, hence it is important to assess the number of mutated reads in other FFPE samples (normal or tumor) that do not have a somatic mutation at the site. We indeed found that some mutations in the FFPE context do appear at noisy sites which are also present in other cases, although typically at much lower allele counts. Accordingly, we applied additional conservative filtering to remove any mutations seen in more than two patients or more than two counts in the normal samples not associated with a mutated tumor. In the tree figures, we annotated both the total number of mutations per clone and the number of mutations that were not in the FFPE context, and there is a column in Supplementary Table 2 stating which mutations are in the FFPE context.

### PoN filtering

To remove sequencing artifacts and frequent germline events, SNVs and indels were filtered using PoNs which includes 8,334 WES normals^63^. Briefly, the panel includes eight values for each site, which describe the percentage of normals, different modes of artifact and the likelihood that the event is a germline event at that site.

### Phylogenetic analysis and subclonal architecture inference

The cancer cell fraction (CCF, represented as a probability density distribution ∈ [0,1]) of individual somatic alterations is estimated using the ABSOLUTE^64^ algorithm (v.1.5) which calculates the sample purity, ploidy and local absolute DNA copy number of each mutation. This CCF distribution represents independent estimates for each somatic event. As multiple somatic events are expected to originate from the same subclone, that is, sharing the same proportion of cancer cells, a clustering algorithm is employed to estimate these proportions and assign events to each of the subpopulations. PhylogicNDT^65^ (v.1.0) is able to identify individual clusters even when the number of mutations per cluster is small. PhylogicNDT performs phylogenetic, tree-building and clustering analysis. In brief, the PhylogicNDT Clustering module employs a multidimensional nonparametric Dirichlet process (DP) on the raw CCF probability density distributions of the somatic variants to learn the underlying clonal structure from the data. The DP is based on the approach where the DP mixing parameter, *α*, is sampled and learned via a Markov Chain Monte Carlo (MCMC) method from the data. The starting assumption of the method is that the posterior CCF distributions are drawn from a mixture of multidimensional distributions with an unknown number of clusters. In this approach, we employ a single, weak prior on *α* parameterized by a negative binomial distribution over the number of clusters *k* (default prior is shape = 3 and scale = 3). It is worth noting that this prior has a minimal effect on the resulting number of clusters. The algorithm is designed as a Gibbs sampler where individual mutations are consecutively assigned to the available clusters through a multinomial distribution representing the likelihood of the mutation belonging to the cluster based on the *n*-dimensional distribution of the cluster CCF position (which is re-estimated after each assignment). At each iteration, there is a probability that the cluster will not retain any mutations and thus will be closed, or that a mutation would open a new cluster. *α* is re-sampled on each DP iteration from a mixture model that depends on *N*, the γ shape and scale parameters, specified prior for *k* and the current *k*. Sampling with such an approach allows the mixing parameter to be learned from the data, rather than requiring its specification upfront. As the number of clusters change through the process, *α* changes accordingly, contributing to the convergence of the method. After completion of the DP-MCMC, the ‘burn-in’ iterations are discarded (first half of the MCMC chain) and the posterior *N*-dimensional CCF distribution of every mutation is estimated based on the average of the CCF distributions along the MCMC chain. The DP also provides a posterior distribution on the total number of clusters. Whenever a fixed number of clusters was required, we selected the least complex likely solution (that is, the lowest number with >10% posterior probability). According to that posterior, the somatic events are then assigned to subclonal cell populations via applying a hierarchical clustering algorithm on their *N*-dimensional posterior CCF distributions, to obtain the CCF distribution of each cluster. Finally, the probability that a mutation belongs to a particular cluster is calculated based on the normalized product of the MCMC CCF distributions associated with the mutation and the posterior CCF distribution of the specific cluster. This uncertainty in cluster membership is later used in constructing the ensemble of phylogenetic trees. Clusters are usually based on >10 mutations, but, if the clusters are minor (for example, based on only a few shared events) and their CCF pattern is very distinct across samples, then a cluster can be formed based on only a few mutations if the CCF confidence intervals (CIs) are defined^66^.

### Construction of phylogenetic trees

The BuildTree component of PhylogicNDT uses the generated posterior distributions on cluster positions and mutation membership to calculate the ensemble of possible trees that support the phylogenetic relationship of the detected cell populations. This algorithm employs an MCMC Gibbs sampler over the branch positions within the tree and parent–child relationships among clones. In each iteration, a subclone can move to a place in the tree according to a multinomial probability calculated based on the pigeon-hole rule (that is, the sum of CCFs of sibling clones cannot exceed the CCF of the parent clone), accounting for the uncertainty in assignment of mutations to subclones. The likelihood of the entire tree is determined by multiplying the pigeon-hole probabilities for all nodes in the tree (that is, parent–children relationships). In each MCMC iteration, the tree likelihoods are used to draw the new location of a single clone. In addition, all mutations are randomly assigned to clones based on the match between their CCF distribution and the clones’ CCF distributions, which are then updated based on the assignment of mutations. This mutation shuffling ensures that the uncertainty in the tree structure also takes into account the uncertainty in mutation assignment. Finally, the MCMC generates a posterior distribution over the possible trees (that is, a ‘forest’ of trees). Clusters with <10% CCF across all analyzed samples from the patient were excluded from the tree. Tumors with <10% purity were excluded from detailed phylogeny analysis.

### Construction of phylogenetic tree diagrams

Phylogenetic tree diagrams throughout the paper are designed in the following way: theoretical cell populations are circles and clones derived from the WES are squares. Any germline *EGFR* mutation found in normal lung tissue is denoted at the top. Branches are configured based on shared and distinct mutations in each clone. Numbers within lineage tracings represent the number of new additional exonic mutations identified in each clone. Numbers in parentheses are exonic mutations that are not in the FFPE context. Additional driver mutations found in tumors are also annotated, including any somatically acquired *EGFR* mutations, with the clones where they were identified in gray boxes. We cannot determine whether clones that share a boxed *EGFR* mutation developed independently or from a shared precursor. Resected tumors are assigned to clones based on their majority population in the layered pie charts shown below the tree. Pie charts below the tree indicate clonal representation within each resected tumor.

### Mutational signature analysis

Mutational signatures were determined using SignatureAnalyzer (v.3ddba7a)^67^. SignatureAnalyzer is a Bayesian NMF (BayesNMF) method that probabilistically infers the number of signatures, *K*, through the automatic relevance determination technique and returns highly interpretable and sparse representations for both underlying mutational signature profiles and patient attributions that strike a balance between data fitting and model complexity. We ran BayesNMF 10,000× using the graphics processing unit implementation with exponential priors for the signature matrixW and activity matrix H and displayed the solution with the maximum posterior^68^. Finally, we compared the identified signatures with those in COSMIC (v.3.2) based on cosine similarity.

### Poly(G) genotype data preprocessing

Generation and analysis of repeat (poly(G)) genotypes were performed as previously described^37–39^. Briefly, 33 poly(G) loci were PCR amplified using primers targeting their flanking sequences. Primer sequences can be found in Naxerova et al.^38^. All reactions were run in duplicate. PCR product length was measured using an ABI 3730xl DNA Analyzer and exported as tab-delimited text files through the Thermo Fisher Scientific Microsatellite Analysis Tool (https://www.thermofisher.com/us/en/home/cloud/all-analysis-modules/sanger-analysis-modules.html). Reactions with intensities <10% of the average intensity for that patient and locus were excluded. If the length distributions of both duplicates were similar (Jensen–Shannon divergence <0.11), the duplicate with higher fluorescence intensity was picked as the representative replicate. At a larger discordance between length distributions, the poly(G) tract was excluded from analysis in all samples of that patient. More details on filtering and quality control of poly(G) genotypes are provided in ref. ^38^. Source data for the poly(G) analysis are available at https://github.com/mblohmer/polyG_egfr_lc.

### Poly(G) genotype data analysis

Amplification of microsatellites produces a characteristic fragment stutter pattern as a result of polymerase slippage during PCR. The mean fragment length at each locus, which represents the genotype of the most recent common ancestor of all sampled cells^69^, was used to simplify this stutter pattern to a single value. Somatic shifts in poly(G) length (mutations) are reported in relation to the normal (germline) sample from each patient. To construct phylogenetic trees using the mean length of poly(G) markers, distance matrices containing all the samples from one patient were constructed using the Manhattan distance. This distance measures the sum of indels among all poly(G) markers in two samples, normalized by the number of poly(G) markers analyzed. As the Manhattan distance simply counts the number of mutations, it scales linearly with the number of cell divisions separating two samples^41^. However, the Manhattan distance is affected by a sample’s purity because the presence of normal cells within a tumor reduces the mean length. Based on the distance matrices, phylogenetic trees were constructed using the neighbor-joining method implemented in the R package ape^70^. Evolutionary distance between two tumors was estimated using Pearson’s correlation coefficient (*r*) between the two vectors of poly(G) marker lengths, as described in detail in ref. ^41^. Only patients in which at least half of all poly(G) markers could be successfully amplified across all samples were considered for this analysis. Samples were analyzed in two batches and only tumors that were analyzed in the same batch were compared with each other. Pearson’s correlation coefficient estimates the fraction of cell divisions in the history of two tumors that they have spent as part of the same lineage. A correlation of 0 means the lineages giving rise to two tumors split at the zygote stage and that they share 0% of their cell divisions, whereas a correlation of 1 means that the tumors’ lineages coincide and they share 100% of their cell divisions. Pearson’s correlation coefficient compares only the direction of mutations, not their magnitude, thus its estimation of evolutionary distance is not affected by purity and mutation rate. To assess the expected correlation value of two unrelated tumors, the distribution of *r* was calculated based on tumors from different patients, across all possible tumor pairs in this cohort, in which at least 15 of the same poly(G) loci were successfully amplified in both samples. CIs for the correlation of poly(guanine) mean lengths were calculated by resampling the poly(G) repeats that were used to calculate it 1,000× with replacement and using the 2.5th to 97.5th percentiles of the results.

### Cell lines

The mouse NIH/3T3 cells were from the American Type Culture collection (cat. no. CRL-1658) and the human NCI-H2228 lung adenocarcinoma cells were a gift from A. Hata (MGH).

### *EGFR*-mutant construct

The WT *EGFR* expression plasmid pHAGE-EGFR was a gift from G. Mills and K. Scott (Addgene plasmid no. 116731)^71^. *EGFR*-mutant constructs containing patient-specific DNA mutations were generated by site-directed mutagenesis (Agilent QuikChange II XL) and confirmed by Sanger sequencing. Overall expression of ectopic *EGFR* constructs was approximately tenfold higher than the level of endogenous expression in human NIC-H2228 cells, a lung cancer cell line that expresses moderate levels of EGFR. These constructs are available on request.

### EGFR in vitro activity assay

EGFR signaling in mouse NIH/3T3 cells, which lack endogenous *EGFR* expression, is measured under three culture conditions: baseline culture (10% serum), 24-h serum starvation (0% serum) and 5 min after addition of EGF (100 ng ml^−1^) to starved cultures. Western blotting for EGFR autophosphorylation at Tyr845 and downstream AKT phosphorylation at Ser473 are used as markers of EGFR activation.

### Western blot analysis

Cells were lysed with radioimmunoprecipitation assay buffer (Sigma-Aldrich, cat. no. R0278) containing protease and phosphatase inhibitors (Life Technologies, cat. nos. A32965 and A32957). Lysate was cleared and western blotted according to standard protocols. The following antibodies were used: EGFR (Cell Signaling Technologies, cat. no. 4267, 1:500 dilution in 5% bovine serum albumin (BSA) in phosphate-buffered saline–Tween (PBST), imaged on LiCor); EGFR pY845 (Cell Signaling Technologies, cat. no. 6963, 1:500 dilution in 5% BSA in PBST, imaged on LiCor); AKT1/2 (Cell Signaling Technologies, cat. no. 9272, 1:1,000 dilution in 5% milk in PBST, imaged on film); AKT pS473 (Cell Signaling Technologies, cat. no. 4060, 1:500 dilution in 5% milk in PBST, imaged on film); and vinculin (Sigma-Aldrich, cat. no. MAB3574, 1:2,000 dilution in 5% BSA in PBST, imaged on LiCor). LiCor Image Studio software v.5.2.5 was used for LiCor western blot quantification and ImageJ v.2.3.0 for film western blot quantification.

### Transformation assays

For soft agar colony formation assays, NIH/3T3 cells stably expressing *RFP*, WT *EGFR* or mutant *EGFR* were suspended in Dulbecco’s modified Eagle’s medium + 10% fetal bovine serum containing 0.4% agarose with no additional EGF for 3 weeks. Colony growth was assayed by staining with 0.2% Crystal Violet in methanol for 10 min, followed by manual counting using ImageJ v.2.3.0.

### DdPCR analysis

DdPCR to detect *EGFR* L858R mutations was performed on TNA extracted from tissue slides. We used the commercially validated probe set for *EGFR* WT and p.L858R c.2573T>G (EGFR HEX and L858R FAM; BioRad Assay, ID dHsaMDV2010021). Samples were prepared following the standard protocol (BioRad). Briefly, 2× ddPCR Supermix for probes (no dUTP) was combined with 20–400 ng of patient DNA, 1× primer/probe mix and 5 U of MseI restriction enzyme (New England Biolabs). After droplet generation, samples were thermocycled with an annealing/extension temperature of 55 °C. BioRad QuantaSoft Analysis Pro software v.1.0 was used for droplet analysis and quantification. Variant allele frequency was calculated as mutant copies per total copies.

### Statistics and reproducibility

No statistical methods were used to predetermine sample sizes, but our sample sizes are similar to those reported in previous publications^15,25^. Tumors with <10% purity were excluded from detailed phylogeny analysis. Reactions with intensities <10% of the average intensity for that patient and locus were excluded from poly(G) analysis. If the length distributions of both duplicates were higher than Jensen–Shannon divergence = 0.11, the poly(G) tract was excluded from analysis in all samples of that patient. Patient 2 was excluded from our poly(G) analysis because fewer than half the poly(G) tracts were successfully amplified across all samples. Some samples evaluated by WES were not assessed via ddPCR or poly(G) analysis because of low sample quantity. No other data were excluded from the analysis. Randomization was not applicable to the present study because the patient samples were retrospectively selected owing to a satisfying set of criteria (more than one EGFR-mutant-resected lung tumor) and were not stratified into treatment arms. The investigators were not blinded to allocation during experiments and outcome assessment. A Kruskal–Wallis test and post-hoc Dunn’s test with Holm–Bonferroni correction were used to calculate the *P* value between groups in Fig. 3g. This test did not assume normality of the data. A two-way analysis of variance (ANOVA) with a Benjamini, Krieger and Yekutieli correction was used to calculate the *q*-value between groups in Fig. 4d–k. A one-tailed, unpaired Student’s *t*-test was used to calculate the *P* value between groups in Fig. 4m,n. The *P* value was not corrected for multiple comparisons. GraphPad Prism v.9.2 and R v.4.1.2 were used for statistical analyses. Data distribution in Fig. 4 was assumed to be normal but this was not formally tested.

### Reporting summary

Further information on research design is available in the Nature Portfolio Reporting Summary linked to this article.

## Supplementary information

Reporting Summary

Supplementary Tables Supplementary Tables 1–7.

## Source data

Source Data Figs. 3 and 4. Fig. 3a Raw ddPCR data. Fig. 3d–f Poly(G) loci correlation between tumors in patient III-1. Fig. 3 Poly(G) relatedness between tumor pairs. Fig. 4d–g,j–m Western blot quantification data, soft agar quantification data. Fig. 4i,n Soft agar quantification data.

Source Data Fig. 4 Unprocessed western blots.

## Data Availability

WES data not already available in the Supplementary Tables that support the findings of the present study have been deposited in dbGaP under accession no. phs003379.v1.p1. Patient III-4 and patient 7 consented to data sharing via direct transfer agreement, available on request to D.A.H. (dhaber@mgh.harvard.edu). Requests will be processed within 2 weeks. Raw poly(G) genotypes are available on GitHub at https://github.com/mblohmer/polyG_egfr_lc. Protein Data Bank (PDB) data referenced in the present study are available at 10.2210/pdb5GTY/pdb and 10.2210/pdb4ZJV/pdb (refs. ^72,73^). All other data supporting the findings of the present study are available from the corresponding author on reasonable request. Source data are provided with this paper.

## Code availability

The code used to analyze the poly(G) genotypes is available on GitHub at https://github.com/mblohmer/polyG_egfr_lc. The code used to analyze the WES data is available at GitHub at https://github.com/getzlab.
