# Supplementary material for: Developmental mosaicism underlying EGFR-mutant lung cancer presenting with multiple primary tumors
Source: Nat Cancer. 2024 Oct 15;5(11):1681–96. doi: 10.1038/s43018-024-00840-y (PMC11584400; doi:10.1038/s43018-024-00840-y)
Supplement: Supplementary file 1 — Reporting Summary [file 43018_2024_840_MOESM1_ESM.pdf]

nature portfolio

Corresponding author(s): Daniel Haber

Last updated by author(s): 09/10/2024

Reporting Summary

Nature Portfolio wishes to improve the reproducibility of the work that we publish. This form provides structure for consistency and transparency in reporting. For further information on Nature Portfolio policies, see our Editorial Policies and the Editorial Policy Checklist.

Statistics

For all statistical analyses, confirm that the following items are present in the figure legend, table legend, main text, or Methods section.

n/a	Confirmed	
☐	☒	The exact sample size (*n*) for each experimental group/condition, given as a discrete number and unit of measurement
☐	☒	A statement on whether measurements were taken from distinct samples or whether the same sample was measured repeatedly
☐	☒	The statistical test(s) used AND whether they are one- or two-sided<br>*Only common tests should be described solely by name; describe more complex techniques in the Methods section.*
☒	☐	A description of all covariates tested
☐	☒	A description of any assumptions or corrections, such as tests of normality and adjustment for multiple comparisons
☐	☒	A full description of the statistical parameters including central tendency (e.g. means) or other basic estimates (e.g. regression coefficient) AND variation (e.g. standard deviation) or associated estimates of uncertainty (e.g. confidence intervals)
☐	☒	For null hypothesis testing, the test statistic (e.g. *F*, *t*, *r*) with confidence intervals, effect sizes, degrees of freedom and *P* value noted<br>*Give P values as exact values whenever suitable.*
☒	☐	For Bayesian analysis, information on the choice of priors and Markov chain Monte Carlo settings
☒	☐	For hierarchical and complex designs, identification of the appropriate level for tests and full reporting of outcomes
☐	☒	Estimates of effect sizes (e.g. Cohen's *d*, Pearson's *r*), indicating how they were calculated

Our web collection on statistics for biologists contains articles on many of the points above.

Software and code

Policy information about availability of computer code

Data collection	LiCor Image Studio v5.2.5 and ImageJ v2.3.0 were used to collect western blotting quantification data. ImageJ v.2.3.0 was used to count soft agar colonies. BioRad QuantaSoft v1.0 was used to collect ddPCR data. Poly-G PCR product length was measured using an ABI 3730xl DNA Analyzer and exported as tab-delimited text files through the ThermoFisher Microsatellite Analysis Tool. RTA v.2.7.3 or later was used to examine flowcells for WES.

Data analysis	Sequence data were analyzed using the Broad Institute's Cancer Genome Analysis WES Characterization Pipeline. This included MuTect (v.1.1.6) for calling somatic single nucleotide variants (sSNVs), Strelka2 (v.2.9.9) for calling small insertions and deletions (indels), deTiN (v.2.0.1) for estimating tumor-in-normal (TiN) contamination, ContEst (v.1.4-437-g6b8a9e1; GATK v.3.7.0) for estimating cross-patient contamination, AllelicCapSeg (v.22) for calling allelic copy number variants, and ABSOLUTE (v.1.5) for estimating tumor purity, ploidy, cancer cell fractions, and absolute allelic copy number. Artifactual variants were filtered out using a token panel-of-normals (PoN) filter, a blat filter, and an oxoG filter. The PhylogicNDT (v.1.0) suite of tools was used to generate posterior distributions on cluster cancer cell fractions and mutation membership to calculate the ensemble of possible trees that support the phylogenetic relationship of detected cell populations. Code for the WES analysis is available here: https://github.com/getzlab. For the poly-G analysis, phylogenetic trees were constructed using the mean length of poly-G markers. Distance matrices containing all the samples from one patient were constructed using the Manhattan distance. This distance measures the sum of insertions and deletions among all poly-G markers in two samples, normalized by the number of poly-G markers analyzed. Because the Manhattan distance simply counts the number of mutations, it scales linearly with the number of cell divisions separating two samples. However, the Manhattan distance is affected by a sample's purity because the presence of normal cells within a tumor reduces the mean length. Based on the distance matrices, phylogenetic trees were constructed using the neighbor-joining method implemented in the R package ape. Code for the poly-G analysis is available here: https://github.com/mblohmer/polyG_egfr_lc. Analysis of western blotting, soft agar, and ddPCR data was performed on GraphPad prism v9.2.

For manuscripts utilizing custom algorithms or software that are central to the research but not yet described in published literature, software must be made available to editors and reviewers. We strongly encourage code deposition in a community repository (e.g. GitHub). See the Nature Portfolio guidelines for submitting code & software for further information.

Data

Policy information about availability of data

All manuscripts must include a data availability statement. This statement should provide the following information, where applicable:

- Accession codes, unique identifiers, or web links for publicly available datasets
- A description of any restrictions on data availability
- For clinical datasets or third party data, please ensure that the statement adheres to our policy

WES data not already available in the Supplementary Tables that support the findings of this study have been deposited in dbGaP under accession number phs003379.v1.p1. Patient III-4 and patient 7 consented to data sharing via direct transfer agreement, available on request to Dr. Daniel Haber (dhaber@mgh.harvard.edu). Requests will be processed within 2 weeks. Raw poly-G genotypes are available on GitHub at https://github.com/mblohmer/polyG_egfr_lc. PDB data referenced in this study is available at https://doi.org/10.2210/pdb5GTY/pdb and https://doi.org/10.2210/pdb4ZJV/pdb72,73. All other data supporting the findings of this study are available from the corresponding author on reasonable request.

Research involving human participants, their data, or biological material

Policy information about studies with human participants or human data. See also policy information about sex, gender (identity/presentation), and sexual orientation and race, ethnicity and racism.

Reporting on sex and gender	The patients were selected based on their presence within a familial cancer pedigree or presentation with multiple primary EGFR mutant cancers. One male and one female familiar cancer patient were studied based on availability of pathology specimens. EGFR mutations are more common in women, compared with men (two-fold), and we selected patients based on available pathology specimens: 10/12 specimens were from female patients. Gender was not considered in the study design based on limited sample availability. Gender reported in Table 1 is self-report by the patient.
Reporting on race, ethnicity, or other socially relevant groupings	We reported genetic ancestry as determined from our patients' medical records, however this data was not used in any analysis.
Population characteristics	We described gender (2M/10F), age (52-85), ancestry (9 European, 2 African, 1 Asian), and smoking history (5 never, 6 former, 1 moderate) for our cohort, however this data was not used in any analysis. Our small cohort, gender distribution and ancestry distribution mean that we may have missed other findings from the less represented groups.
Recruitment	Participants were recruited to deposit their tissue in an MGH biobank for subsequent deidentified study at time of surgery. Patients were retrospectively selected for this study by having more than one resected primary lung cancer which were geographically distinct on CT imaging AND have at least one known EGFR mutation in one of their primary lung cancers. An additional cohort of 6 patients within these parameters who were light or never smokers were selected out for a second cohort. The MGH patient population is not representative of the surrounding area, which may have introduced bias in the demographics of patients included in this study but would not have impacted the results. Our focus on patients with EGFR-mutant lung cancers biased towards never or light smokers, therefore our results are only relevant to this population and not to the broader NSCLC population.
Ethics oversight	Two protocols were used for this study, both reviewed and approved by the Dana Farber/Harvard Cancer Center (DF/HCC) Institutional Review Board, which oversees clinical cancer protocols for all Harvard institutions including MGH. For the tumor blocks from sporadic lung cancer cases, the approved protocol DF/HCC 13-416 includes permission for molecular analysis, privacy of results, and publication of deidentified results. For the T790M-familial pedigree, the cases were initially collected under protocol DF/HCC 94-138 at the time of the initial publication [Bell et al., Nature Genetics, 2005, PMID 16258541)] and reconsented under protocol DF/HCC 13-416 for the current study. Under both protocols all participants provided written informed consent for collection of tumor and tissue specimens and clinical information for inclusion in a tissue repository for future research including DNA and RNA sequencing except where noted, and written informed consent for sharing of clinical information in deidentified publications.

Note that full information on the approval of the study protocol must also be provided in the manuscript.

Field-specific reporting

Please select the one below that is the best fit for your research. If you are not sure, read the appropriate sections before making your selection.

☒ Life sciences ☐ Behavioural & social sciences ☐ Ecological, evolutionary & environmental sciences

For a reference copy of the document with all sections, see nature.com/documents/nr-reporting-summary-flat.pdf

Life sciences study design

All studies must disclose on these points even when the disclosure is negative.

Sample size	No sample size calculations were performed in this study. For the soft agar assay, the effect size was large enough that n=3 was sufficient to capture the change. This is consistent with our previous experience with this assay (see Godin-Heymann et al Cancer Research 2007). For the western blotting assays, we have extensive experience with the EGFR activation assay to indicate that there is high variability in the quantified data (see Lynch et al NEJM 2004; Godin-Heymann et al Cancer Research 2007). Combined with our stated modest effect size, n=10 was necessary to accurately report on the phenomenon. For the ddPCR, WES, and poly-G analysis, the sample size was all samples available from our patient cohort.
Data exclusions	Patient 2 was excluded from our poly-G analysis because their samples did not pass quality control. Some samples evaluated by WES were not assessed via ddPCR or poly-G analysis due to low sample quantity.
Replication	Reproducibility of our cell and molecular biology findings was evaluated through multiple samples. In all cases, n indicates independent replicates. All attempts at replication are reported in the paper. Reproduction of our overall findings on another patient cohort is beyond the scope of this paper.
Randomization	Randomization was not applicable to this study because the patient samples were retrospectively selected due to satisfying a set of criteria (more than one EGFR-mutant resected lung tumor) and were not stratified into treatment arms.
Blinding	Blinding is not relevant to this study as no group allocation was performed.

Reporting for specific materials, systems and methods

We require information from authors about some types of materials, experimental systems and methods used in many studies. Here, indicate whether each material, system or method listed is relevant to your study. If you are not sure if a list item applies to your research, read the appropriate section before selecting a response.

Materials & experimental systems

n/a	Involved in the study	
☐	☒	Antibodies
☐	☒	Eukaryotic cell lines
☒	☐	Palaeontology and archaeology
☒	☐	Animals and other organisms
☒	☐	Clinical data
☒	☐	Dual use research of concern
☒	☐	Plants

Methods

n/a	Involved in the study	
☒	☐	ChIP-seq
☒	☐	Flow cytometry
☒	☐	MRI-based neuroimaging

Antibodies

Antibodies used	EGFR (Cell Signaling Technologies #4267, multiple lots, 1:500 dilution); EGFR pY845 (Cell Signaling Technologies #6963, multiple lots, 1:500 dilution); Akt1/2 (Cell Signaling Technologies #9272, multiple lots, 1:1000 dilution); Akt pS473 (Cell Signaling Technologies #4060, multiple lots, 1:500 dilution); Vinculin (Sigma Aldrich MAB3574 clone VIIF9, multiple lots, 1:2000 dilution).
Validation	EGFR and pEGFR antibodies were validated in our study by comparing performance on NIH3T3 cells not expressing and overexpressing EGFR. The pAkt antibody was validated in our study by showing the established response to EGF treatment of cells expressing WT EGFR. The Akt antibody was validated by CST through western blot analysis of extracts from CHO cells, transfected with non-targeted (-) or SignalSilence® Akt siRNA I (+) siRNA. The vinculin antibody was validated by comparison with Ponceau S staining.

Eukaryotic cell lines

Policy information about cell lines and Sex and Gender in Research

Cell line source(s)	The mouse NIH/3T3 cells were from ATCC (cat #CRL-1658) and the human NCI-H2228 lung adenocarcinoma cells were a gift from Dr. Aaron Hata (Mass General Hospital)
Authentication	These cell lines were not authenticated.
Mycoplasma contamination	These cell lines and all lines derived from them tested negative for mycoplasma.
Commonly misidentified lines (See ICLAC register)	Neither of these are commonly misidentified lines.
