# Supplementary material for: Developmental mosaicism underlying EGFR-mutant lung cancer presenting with multiple primary tumors
Source: Nat Cancer. 2024 Oct 15;5(11):1681–96. doi: 10.1038/s43018-024-00840-y (PMC11584400; doi:10.1038/s43018-024-00840-y)
Supplement: Supplementary file 4 — Unprocessed western blots. [file 43018_2024_840_MOESM4_ESM.pdf]

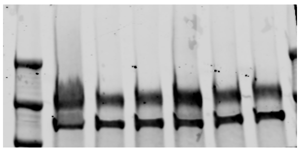

Fig 4d anti-EGFR and anti-vinculin

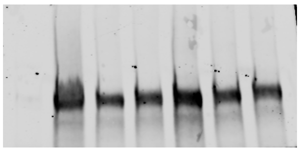

Fig 4d only anti-EGFR (same as above but no vinculin, no ladder)

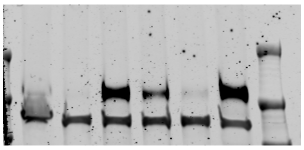

Fig 4d anti-pY845 and anti-vinculin

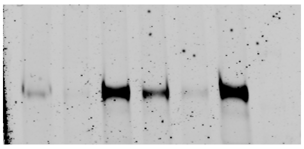

Fig 4d only anti-pY45 (same as above but no vinculin, no ladder)

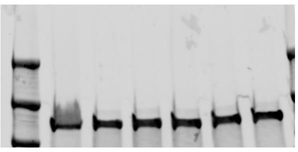

Fig 4d anti vinculin

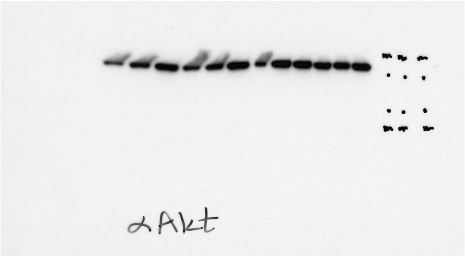

Fig 4e anti-Akt

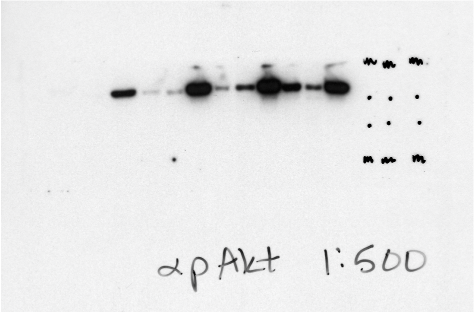

Fig 4e anti-pAkt

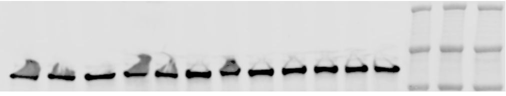

Fig 4e anti-vinculin

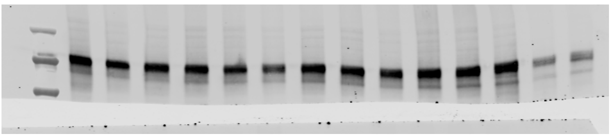

Fig 4h anti-EGFR

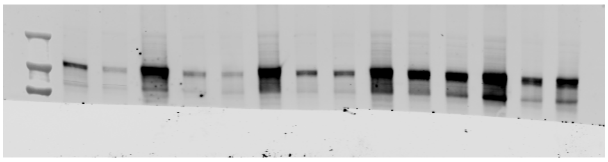

Fig 4h anti-pY845

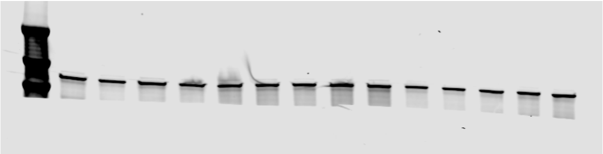

Fig 4h anti-vinculin

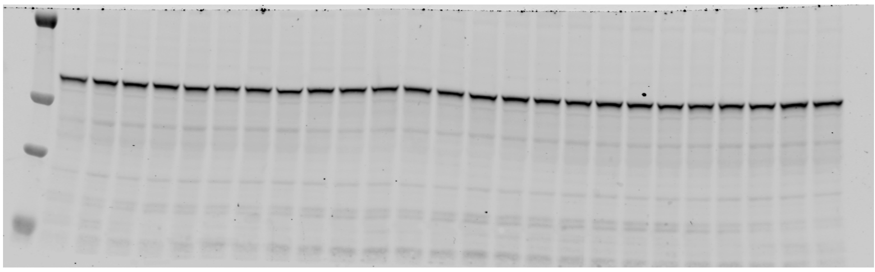

Fig 4i anti-Akt

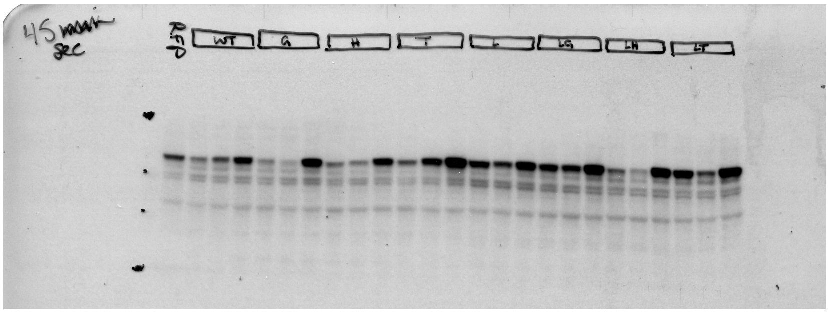

Fig 4i anti-pAkt

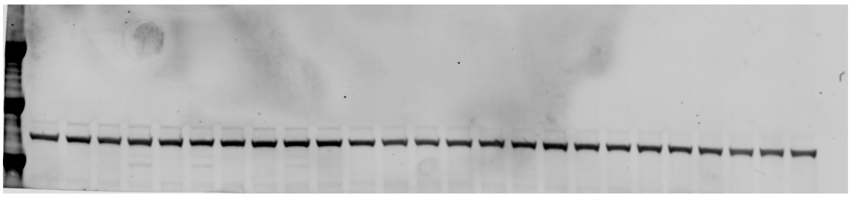

Fig 4i anti-vinculin
